# Supplementary material for: MetaRibo-Seq measures translation in microbiomes
Source: Nat Commun. 2020 Jun 29;11:3268. doi: 10.1038/s41467-020-17081-z (PMC7324362; doi:10.1038/s41467-020-17081-z)
Supplement: Supplementary file 10 — Supplementary Data 7 [file 41467_2020_17081_MOESM10_ESM.zip › File2/Confidence_High_Taxonomy/299379_out.krona.html]

Javascript must be enabled to view this page.

members
magnitude
magnitudeUnassigned
count
unassigned
taxon
rank

299379\_out

2

superkingdom
2
2

2
phylum
1239

1263019
species
1

SRS078242\_contig\_number\_contig-100\_3109.88443

91061
class
1

1385
order
1

1
family
186817

1
1386
genus

species
1882761

SRS058070\_contig\_number\_21033
1
